# Interleukin-37 is increased in adult-onset Still’s disease and associated with disease activity

**DOI:** 10.1186/s13075-018-1555-6

**Published:** 2018-03-22

**Authors:** Huihui Chi, Dongzhou Liu, Yue Sun, Qiongyi Hu, Honglei Liu, Xiaobing Cheng, Junna Ye, Hui Shi, Yufeng Yin, Mengru Liu, Xinyao Wu, Zhuochao Zhou, Jialin Teng, Chengde Yang, Yutong Su

**Affiliations:** 1 Department of Rheumatology and Immunology, Ruijin Hospital, Shanghai Jiao Tong University School of Medicine, No. 197 Ruijin Second Road, Shanghai, 200025 China; 2 Department of Rheumatology and Immunology, Shenzhen People’s Hospital, the Second Clinical Medical College of Jinan University, Shenzhen, China

**Keywords:** Adult-onset Still’s disease, IL-37, Cytokines, Peripheral blood mononuclear cells

## Abstract

**Background:**

Interleukin (IL)-37 has been known to play an immunosuppressive role in various inflammatory disorders, but whether it participates in the regulation of pathogenesis of adult-onset Still’s disease (AOSD) has not been investigated. In this study, we examined serum IL-37 levels and their clinical association with AOSD, and we explored the anti-inflammatory effects of IL-37 on peripheral blood mononuclear cells (PBMCs) from patients with AOSD.

**Methods:**

Blood samples were collected from 62 patients with AOSD and 50 healthy control subjects (HC). The serum IL-37 levels were determined using an enzyme-linked immunosorbent assay (ELISA). The correlations of serum IL-37 levels with disease activity, laboratory values, and inflammatory cytokines in AOSD were analyzed by Spearman’s correlation test. The correlations between serum IL-37 levels and clinical manifestations were analyzed by Mann-Whitney *U* test. PBMCs from ten patients with AOSD were stimulated with recombinant human IL-37 protein, and expression levels of tumor necrosis factor (TNF)-α, IL-6, IL-10, IL-1β, and IL-18 were determined by qRT-PCR and ELISA.

**Results:**

A significantly higher IL-37 protein level was observed in patients with AOSD than in HC. Serum IL-37 levels correlated with systemic score, laboratory values, IL-1β, IL-18, and IL-10 in patients with AOSD. The expression levels of IL-37 were closely related to the patients with AOSD who also had fever, skin rash, lymphadenopathy, splenomegaly, myalgia, and arthralgia. Moreover, the production of proinflammatory cytokines such as IL-6, IL-1β, TNF-α, and IL-18 in PBMCs from patients with AOSD was obviously attenuated after recombinant human IL-37 stimulation.

**Conclusions:**

Increased expression of IL-37 and its positive correlation with disease activity suggest its involvement in AOSD pathogenesis. More importantly, IL-37 inhibits the expression of proinflammatory cytokines in PBMCs from patients with AOSD, indicating the potential anti-inflammatory role of IL-37 in AOSD. Thus, IL-37 may be a novel disease activity biomarker and research target in AOSD.

**Electronic supplementary material:**

The online version of this article (10.1186/s13075-018-1555-6) contains supplementary material, which is available to authorized users.

## Background

Adult-onset Still’s disease (AOSD) is a rare systemic inflammatory disorder characterized by spiking fever, evanescent rash, arthralgia or arthritis, sore throat, lymphadenopathy, hepatosplenomegaly, and myalgia [1–4]. Although the pathogenic mechanism of AOSD is still largely unknown, multiple factors, including a predisposing genetic background, viral infections, and aberrant immune response, have been suggested to be involved in the development of this disease [4–7].

Proinflammatory cytokines, such as interleukin (IL)-1β, IL-6, IL-18, tumor necrosis factor-α (TNF-α), and interferon-γ (IFN-γ), have been found to be elevated during AOSD and are thought to be involved in the pathogenesis of AOSD [8–13]. IL-18 and IL-1β, two proinflammatory cytokines processed through the inflammasome machinery, are key factors in the pathogenesis of AOSD; they cause IL-6 and Th1 cytokine secretion as well as natural killer cell dysregulation leading to macrophage activation [3]. A growing body of clinical trials support the hypothesis that blocking these cytokines could partly relieve inflammatory symptoms of AOSD and reduce disease severity, such as TNF-α blockers and IL-1β and IL-6 antagonists [8, 14, 15]. Increasing evidence regarding the efficacy of IL-1 inhibitors such as anakinra (IL-1 receptor antagonist) and canakinumab (monoclonal anti-IL-1β antibody) has been collected from patients with refractory AOSD [16, 17]. Indeed, there is accumulating evidence that the clinical course of AOSD can be divided into systemic monophasic, polycyclic, and chronic articular patterns or a systemic pattern and an articular pattern, based on cytokine profile, clinical presentation, and outcome [3, 8, 18–20]. Anakinra may prove to be more effective in patients with the systemic disease pattern than in the chronic articular one, especially in those patients experiencing severe complications such as macrophage activation syndrome [21, 22]. In the event of an insufficient response to anakinra, canakinumab can be considered because of its longer half-life. Both agents have been reported to be effective in AOSD [23]. On the basis of these studies, IL-1 inhibitors seem to represent the best biologic disease-modifying antirheumatic drugs available and should be considered the first-line biologic treatment for patients not responding to conventional treatment. Recent studies have indicated that IL-37 downregulated the expression of proinflammatory cytokines in chronic inflammatory diseases such as systemic lupus erythematosus (SLE) [24], rheumatoid arthritis (RA) [25–27], and ankylosing spondylitis (AS) [28], suggesting that IL-37 might abrogate proinflammatory cytokine production to reduce inflammatory responses in AOSD.

IL-37, a newly discovered member of the IL-1 family, has been identified as a natural inhibitor of immune responses [29]. The IL-37 expression level was markedly upregulated in peripheral blood monocular cells (PBMCs), epithelial cells, macrophages, dendritic cells (DCs), and T cells following the stimulation of proinflammatory cytokines, including IL-1β, IL-18, TNF-α, and IFN-γ, and the Toll-like receptor (TLR) ligand lipopolysaccharide (LPS) [30–32]. The anti-inflammatory properties of IL-37 were first revealed by transfection of human IL-37 into mouse macrophages, resulting in suppression of TLR-induced proinflammatory cytokines [31]. Unlike other IL-1 family members, a homolog gene for IL-37 has not been identified in the mouse. Nevertheless, transgenic mice expressing the human IL-37 gene were generated (IL-37-transgenic [IL37-tg]) and have extensively revealed IL-37 anti-inflammatory properties [29]. IL37-tg mice exhibited a remarkable protection of spinal cord contusion injury [33]. Similarly, recombinant IL-37 treatment in wild-type mice was effective in inflammatory arthritis [27] and revealed a property of IL-37 that induced metabolic reprogramming and limited metabolic costs of inflammation, thus ameliorating inflammation-induced fatigue [34]. Another study demonstrated that IL-37 inhibited nuclear factor-κB (NF-κB) activation induced by oxidized low-density lipoprotein in human aortic valve cells [35]. In recent years, the inhibitory activity of IL-37 has also been investigated in autoimmune diseases. Upregulated expression of IL-37 in serum has been reported in many inflammation-related disorders, such as SLE, RA, AS, and Graves’ disease [24–28, 30, 36]. Concomitantly, upregulated IL-37 significantly suppresses the production of proinflammatory cytokines in PBMCs from subjects with SLE, RA, and AS in vitro [24–26, 28]. However, whether immunosuppressive features of IL-37 contribute to the pathogenesis of autoinflammatory diseases such as AOSD is still unclear.

In the present study, we investigated the expression of IL-37 in the serum of patients with AOSD. In addition, we determined the correlation of serum IL-37 levels with disease activity, laboratory parameters, inflammatory cytokines, and disease manifestations in patients with AOSD. We further studied the effect of IL-37 on cytokine production in PBMCs from patients with AOSD.

## Methods

### Patients and healthy control subjects

A total of 62 patients with AOSD were enrolled in this study. Patients with AOSD were diagnosed according to Yamaguchi et al.’s criteria [37] after infections, malignancy, and other autoimmune diseases were excluded. Fifty age- and sex-matched volunteers with no history of autoimmune, rheumatic, or other diseases were recruited as healthy control subjects (HC). The study was performed in accordance with the Declaration of Helsinki and the principles of good clinical practice. Biological samples were obtained under a protocol approved by the Institutional Research Ethics Committee of Ruijin Hospital (identifier 2016-62), Shanghai, China. Informed consent was obtained from recruited subjects.

The medical histories and clinical characteristics, including those identified during a physical examination, of all subjects were collected. Serum samples of the patients with active AOSD were collected before steroid or synthetic disease-modifying antirheumatic drug treatment. Follow-up samples were collected from ten patients with active AOSD after resolution of disease activity. Laboratory findings, including a complete blood count, erythrocyte sedimentation rate (ESR), C-reactive protein (CRP), rheumatoid factor, antinuclear antibody, ferritin, and liver function tests were reviewed. AOSD disease activity was assessed according to the systemic disease score method widely accepted and used [38, 39], which scores disease activity from 0 to 12 and adds 1 point for each of the following manifestations: fever, evanescent rashes, sore throat, arthritis, myalgia, pleuritis, pericarditis, pneumonitis, lymphadenopathy, hepatomegaly or abnormal liver function tests, elevated leukocyte count > 15,000/μl, and serum ferritin > 3000 μg/L. Patients with AOSD were considered to have clinically active disease if they had fever and/or inflammatory arthralgia/arthritis and/or any suggestive cutaneous lesions and/or sore throat. Their disease was otherwise considered inactive [40].

### Blood collection and PBMC isolation

Venous blood samples were obtained. PBMCs were isolated from patients with AOSD and HC using Lymphoprep™ (STEMCELL Technologies, Vancouver, BC, Canada) under sterile conditions following the manufacturer’s instructions [41]. The collected cells were used for cell cultures or frozen at − 80 °C until RNA extraction. Serum samples were stored at − 80 °C until cytokines were determined.

### Cell culture

Whole-blood PBMCs were cultured in RPMI 1640 medium (Thermo Fisher Scientific, Waltham, MA, USA) with 100 μg/ml streptomycin (Thermo Fisher Scientific), 100 IU/ml penicillin, and 10% FBS (Thermo Fisher Scientific) as culture medium in a humidified atmosphere of 5% CO_2_ at 37 °C. PBMCs were stimulated with or without recombinant human IL-37 (rhIL-37, catalogue number 1975-IL-025; R&D Systems, Minneapolis, MN, USA) at various concentrations for 24 h, then incubated further with 1 μg/ml LPS (Sigma-Aldrich, St. Louis, MO, USA) for 4 h. Total RNA was extracted [42], and cytokine transcription was analyzed by qRT-PCR. To determine cytokine protein expression in PBMCs, cells were stimulated with or without rhIL-37 at 100 ng/ml for 24 h, then incubated further with LPS (1 μg/ml) for 8 h, and culture supernatants were harvested and frozen at − 80 °C for later cytokine analysis by enzyme-linked immunosorbent assay (ELISA).

### RNA extraction and qRT-PCR

Total RNA was extracted with TRIzol reagent (Thermo Fisher Scientific) according to the manufacturer’s instructions. For messenger RNA (mRNA) reverse transcription, 1 μg of the total RNA was converted into complementary DNA in a 20-μl reaction volume using a reverse transcription kit (Promega, Madison, WI, USA) following the manufacturer’s instructions. PCR primers used for qRT-PCR were as follows: human IL-37: forward 5′-AGTGAGGTCAGCGATTAGGA-3′ and reverse 5′-TTTTAGTGAGCAGGTTTGGT-3′; human glyceraldehyde 3-phosphate dehydrogenase (GAPDH): forward 5′- CTGGGCTACACTGAGCACC-3′ and reverse 5′-AAGTGGTCGTTGAGGGCAATG-3′; human IL-6: forward 5′-ACTCACCTCTTCAGAACGAATTG-3′ and reverse 5′- CCATCTTTGGAAGGTTCAGGTTG-3′; human IL-18: forward 5′- TCTTCATTGACCAAGGAAATCGG-3′ and reverse 5′- TCCGGGGTGCATTATCTCTAC-3′; human TNF-α: forward 5′- GAGGCCAAGCCCTGGTATG-3′ and reverse 5′-CGGGCCGATTGATCTCAGC-3′; human IL-10: forward 5′-GACTTTAAGGGTTACCTGGGTTG-3′ and reverse 5′-TCACATGCGCCTTGATGTCTG-3′; human IL-1β: forward 5′- ATGATGGCTTATTACAGTGGCAA-3′ and reverse 5′-GTCGGAGATTCGTAGCTGGA-3′. qPCR was performed using a LightCycler 480 real-time PCR system (Roche Applied Science, Indianapolis, IN, USA). RNA samples were normalized to control housekeeping genes (human GAPDH), and relative mRNA levels of target genes were calculated using the 2^−ΔΔCt^ comparative cycle threshold method.

### Cytokine assessment

Serum IL-37 levels and cell culture supernatant TNF-α, IL-1β, IL-18, IL-6, and IL-10 levels were determined by ELISA following the manufacturer’s instructions. Serum IL-37 levels were quantified using commercial ELISA reagent kits purchased from AdipoGen Life Sciences (San Diego, CA, USA). Detection of the TNF-α, IL-1β, IL-18, IL-6, and IL-10 in the cell culture supernatant was accomplished using an ELISA kit (R&D Systems). Serum TNF-α, IL-1β, IL-18, IL-6, and IL-10 levels were measured using the Meso Scale Discovery electrochemiluminescence assay (MSD, Rockville, MD, USA).

### Statistical analysis

Data were expressed as mean ± SD and analyzed by Prism version 5.00 software (GraphPad Software, La Jolla, CA, USA). Spearman’s correlation test was used to assess the association between serum IL-37 levels and different variables. Comparisons between groups were made using the nonparametric Mann-Whitney *U* test. The Wilcoxon signed-rank test was used to compare IL-37 levels in patients who underwent follow-up serum sampling. The differences were considered significant at *P <* 0.05.

## Results

### Serum IL-37 levels were higher in patients with AOSD compared with healthy control subjects

**Fig. 1:**
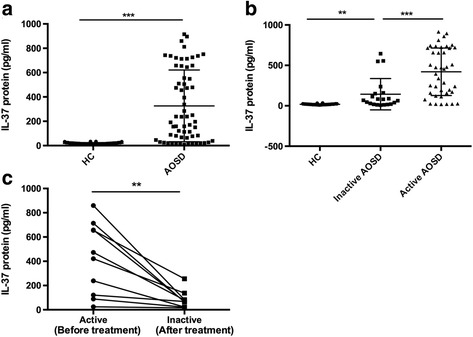
The expression of interleukin (IL)-37 protein levels in patients with adult-onset Still’s disease (AOSD). **a** Comparison of IL-37 protein levels between patients with AOSD (*n* = 62) and healthy control subjects (HC) (*n* = 50). **b** Comparison of IL-37 protein levels among patients with AOSD with active and inactive disease activity (active [*n* = 41] versus inactive [*n* = 21]) as well as in HC (*n* = 50). **c** Change in IL-37 protein levels in ten patients with active AOSD according to disease activity. Serum IL-37 protein levels were determined by enzyme-linked immunosorbent assay. Each symbol represents an individual patient with AOSD and an HC. *Horizontal lines* indicate median values. The data represent the mean ± SD. ** *P* < 0.01, *** *P* < 0.001 by Student’s *t* test in (**a**) and (**b**). The Wilcoxon signed-rank test was used to perform statistical analysis in (**c**)

The serum IL-37 levels of 62 patients with AOSD and 50 age- and sex-matched HC were measured by ELISA. Patients with AOSD and HC did not have significant differences in terms of mean age or sex distribution (Table 1). As shown in Fig. 1a, patients with AOSD had significantly higher serum IL-37 protein levels than HC, indicating that IL-37 probably participated in the pathogenesis of AOSD. We next investigated whether IL-37 was related to disease activity in patients with AOSD. We divided patients with AOSD into active and inactive groups according clinical manifestations mentioned above (Table 1). As seen in Fig. 1b, significant differences in IL-37 protein levels were found between patients with active versus inactive disease activity. Moreover, patients with inactive disease displayed higher serum IL-37 protein levels than HC. During serial follow-up, IL-37 showed a significant decrease in the inactive phase (Fig. 1c). Thus, we speculated that IL-37 was probably associated with AOSD disease activity.

**Table 1:** Clinical characteristics of patients at enrollment

	AOSD (*n* = 62)		
	Active (*n* = 41)	Inactive (*n* = 21)	HC (*n* = 50)
Age, years	43.4 ± 14.1	37.5 ± 14.2	38.6 ± 11.7
Sex, F/M	31/10	15/6	37/13
Duration, months	2.4 ± 2.7	13.7 ± 13.0	
Clinical features			
Fever	38 (92.7)	0	
Sore throat	30 (73.2)	0	
Skin rash	32 (78.0)	2 (9.5)	
Lymphadenopathy	30 (73.2)	2 (9.5)	
Splenomegaly	15 (36.6)	0	
Hepatomegaly	1 (2.4)	0	
Pericarditis	8 (19.5)	0	
Pleuritis	10 (24.4)	0	
Myalgia	14 (34.1)	0	
Arthralgia	37 (90.2)	0	
Arthritis	17 (41.5)	1 (4.8)	
Systemic score	6.9 ± 2.0	0.2 ± 0.5	
Laboratory markers			
Hemoglobin, g/L	110.2 ± 25.8	130.0 ± 16.5	
Leukocytes, 10^9^/L	17.0 ± 5.5	8.1 ± 2.5	
Platelets, 10^9^/L	285.7 ± 119.6	248.9 ± 74.9	
ESR, mm/h	73.2 ± 27.1	17.0 ± 27.9	
CRP, mg/L	91.5 ± 54.8	13.5 ± 16.6	
ALT, U/L	63.1 ± 58.9	27.1 ± 12.0	
AST, U/L	49.1 ± 20.1	31.1 ± 10.3	
Ferritin, ng/ml	9665.2 ± 4692.7	181.8 ± 162.6	
ANA positivity	6 (14.6)	1 (4.8)	
RF positivity	2 (4.9)	0	
Treatments			
Steroid- and sDMARD-naïve	41 (100)	2^a^ (9.5)	
Low-dose steroid monotherapy	0	0	
High-dose steroid monotherapy	0	0	
sDMARD(s)	0	6 (28.6)	
Combination therapy, steroids + sDMARD(s)	0	13 (61.9)	

*Abbreviations: AOSD* Adult-onset Still’s disease, *HC* Healthy control, *ESR* Erythrocyte sedimentation rate, *CRP* C-reactive protein, *AST* Aspartate transaminase, *ALT* Alanine transaminase, *ANA* Antinuclear antibody, *RF* Rheumatoid factor, *sDMARD* Synthetic disease-modifying antirheumatic drug

Low dose of steroids was defined as ≤ 0.5 mg/kg/day of prednisone; high dose of steroids was defined as > 0.5 mg/kg/day of prednisone

All values are presented as number (percent) or mean ± SD

^a^Drug withdrawal

### Correlation between IL-37 levels and AOSD disease activity score as well as laboratory values

To further survey the relationship between serum IL-37 protein levels and disease activity, we next detected the correlations between IL-37 and AOSD disease activity score as well as laboratory values such as leukocytes, ESR, ferritin, CRP, alanine transaminase, and aspartate transaminase. A significantly positive correlation was observed between serum IL-37 levels and systemic score (*r* = 0.4152, *P* = 0.0008), as well as leukocytes (*r* = 0.3019, *P* = 0.018), ESR (*r* = 0.3071, *P* = 0.0161), ferritin (*r* = 0.5303, *P* = 0.0001), and CRP (*r* = 0.3573, *P* = 0.0047) (Table 2). No significant correlations were found between serum IL-37 levels and liver function.

**Table 2:** Correlation of serum IL-37 levels with disease activity score and laboratory values

Disease activity markers	Correlation coefficient *r* (*P* value)
Leukocytes	0.3019 (0.018)
ESR	0.3071 (0.0161)
CRP	0.3573 (0.0047)
AST	0.1366 (0.3883)
ALT	− 0.0424 (0.7562)
Ferritin	0.5303 (0.0001)
Systemic score	0.4152 (0.0008)

*Abbreviations: ESR* Erythrocyte sedimentation rate, *CRP* C-reactive protein, *AST* Aspartate transaminase, *ALT* Alanine transaminase

These data were analyzed using Spearman’s correlation coefficient

The systemic score described by Rau et al. [39] assigns a score from 0 to 12 and adds 1 point for each of the following manifestations: fever, evanescent rashes, sore throat, arthritis, myalgia, pleuritis, pericarditis, pneumonitis, lymphadenopathy, hepatomegaly or abnormal liver function tests, elevated leukocyte count > 15,000/μl, and serum ferritin > 3000 μg/L

### Associations of serum IL-37 levels with inflammatory cytokines levels

**Fig. 2:**
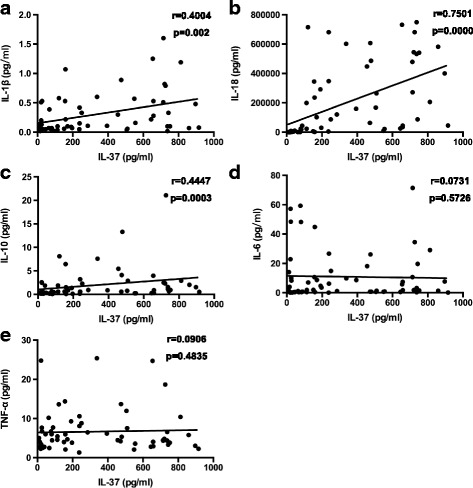
Associations between serum interleukin (IL)-37 levels and inflammatory cytokines in patients with adult-onset Still’s disease. Serum IL-37 levels were positively correlated with IL-1β (**a**), IL-18 (**b**), and IL-10 (**c**) respectively except for IL-6 (**d**) and TNF-α (**e**). Each symbol represents an individual patient. The correlations were evaluated with Spearman’s nonparametric test. *P* < 0.05 represents a significant difference. *TNF-α* Tumor necrosis factor-α

Published studies have demonstrated that proinflammatory cytokines IL-1β, TNF-α, IL-6, IL-18, and IL-17 play an important role in promoting AOSD disease development [14, 19, 40, 43, 44]. Consistent with these findings, we also demonstrated that the levels of serum IL-1β, IL-1Rα, TNF-α, soluble tumor necrosis factor receptor, IL-6, IL-18, and IL-17 were significantly higher in patients with active AOSD than in those with inactive AOSD and in HC. Moreover, serum TNF-α, IL-1Rα, and IL-18 were still higher in the inactive group than in HC (*see* Additional file 1: Figure S1). To assess the potential relationships of serum IL-37 levels with the levels of the proinflammatory cytokines mentioned above in patients with AOSD, the correlations between IL-37 and IL-1β, TNF-α, IL-6, and IL-18 were analyzed by Spearman’s correlation test. The results indicated that the concentrations of serum IL-37 were positively correlated with the concentrations of serum IL-1β (*r* = 0.4004, *P* = 0.002) (Fig. 2a) and IL-18 (*r* = 0.7501, *P* = 0.0000) (Fig. 2b). IL-10, an anti-inflammatory cytokine, showed higher expression levels in patients with active AOSD than in those with inactive AOSD and in HC (*see* Additional file 1: Figure S1). Interestingly, the levels of the anti-inflammatory cytokine IL-37 were positively correlated with IL-10 (*r* = 0.4447, *P* = 0.0003) (Fig. 2c).

### Association of serum IL-37 protein levels with clinical features in patients with AOSD

To assess associations between serum IL-37 protein levels and clinical manifestations in patients with AOSD, serum IL-37 protein levels were compared among patients with and those without certain clinical features. When the serum IL-37 levels were analyzed with regard to the manifestations of AOSD, the patients who had fever had a higher serum IL-37 level (428.42 ± 291.4 pg/ml) than the patients who did not have fever (165.33 ± 221.06 pg/ml) (*P* = 0.0003) (Table 3), and the patients who had a skin rash had a higher level (416.33 ± 308.19 pg/ml) than those who did not have a rash (217.60 ± 239.06 pg/ml) (*P* = 0.0041) (Table 3). Moreover, the patients who had sore throat, lymphadenopathy, splenomegaly, myalgia, and arthralgia had a higher level of IL-37 than those who did not have these symptoms (Table 3). Together, these data demonstrate that serum IL-37 protein levels correlated significantly with clinical features in patients with AOSD. On the basis of the proposed classification of disease courses of AOSD [45], we had follow-up of the patients with AOSD for at least 1 year, and we classified them into three kinds of courses: monophasic (*n* = 39), polycyclic (*n* = 20), and chronic articular patterns (*n* = 3). The serum IL-37 levels were comparable between these three groups (Additional file 1: Figure S2b). We also divided our patients with AOSD into those with a systemic pattern (*n* = 59) and those with a chronic articular pattern (*n* = 3). Again, the serum IL-37 levels were comparable between these two groups (Additional file 1: Figure S2a).

**Table 3:** Comparison of the serum IL-37 levels according to disease manifestations in 62 patients with adult-onset Still’s disease

Manifestations	Serum IL-37 levels		*P* value
Fever	(+), *n* = 38	428.42 ± 291.40	0.0003
	(−), *n* = 24	165.33 ± 221.06	
Sore throat	(+), *n* = 30	419.32 ± 294.61	0.0127
	(−), *n* = 32	239.64 ± 270.42	
Skin rash	(+), *n* = 34	416.33 ± 308.19	0.0041
	(−), *n* = 28	217.60 ± 239.06	
Lymphadenopathy	(+), *n* = 32	436.64 ± 288.01	0.0005
	(−), *n* = 30	209.18 ± 256.72	
Hepatomegaly	(+), *n* = 1	156.63	0.7586
	(−), *n* = 61	329.37 ± 295.93	
Splenomegaly	(+), *n* = 15	477.52 ± 311.97	0.015
	(−), *n* = 47	278.41 ± 274.67	
Pericarditis	(+), *n* = 8	432.69 ± 301.64	0.1824
	(−), *n* = 54	310.86 ± 292.79	
Pneumonia	(+), *n* = 20	379.57 ± 310.59	0.3544
	(−), *n* = 42	301.34 ± 286.62	
Pleuritis	(+), *n* = 10	479.63 ± 336.37	0.0978
	(−), *n* = 52	297.15 ± 279.57	
Myalgia	(+), *n* = 14	449.08 ± 222.11	0.0211
	(−), *n* = 48	290.85 ± 304.96	
Arthritis	(+), *n* = 18	427.91 ± 324.91	0.1068
	(−), *n* = 44	285.13 ± 274.02	
Arthralgia	(+), *n* = 38	405.41 ± 297.41	0.0026
	(−), *n* = 24	201.77 ± 246.81	

*IL* Interleukin

Serum IL-37 levels are shown as mean ± SD, and differences between two groups were analyzed using the Mann-Whitney *U* test for nonparametric data

### IL-37 inhibits the productions of inflammatory cytokines in PBMCs of patients with AOSD

**Fig. 3:**
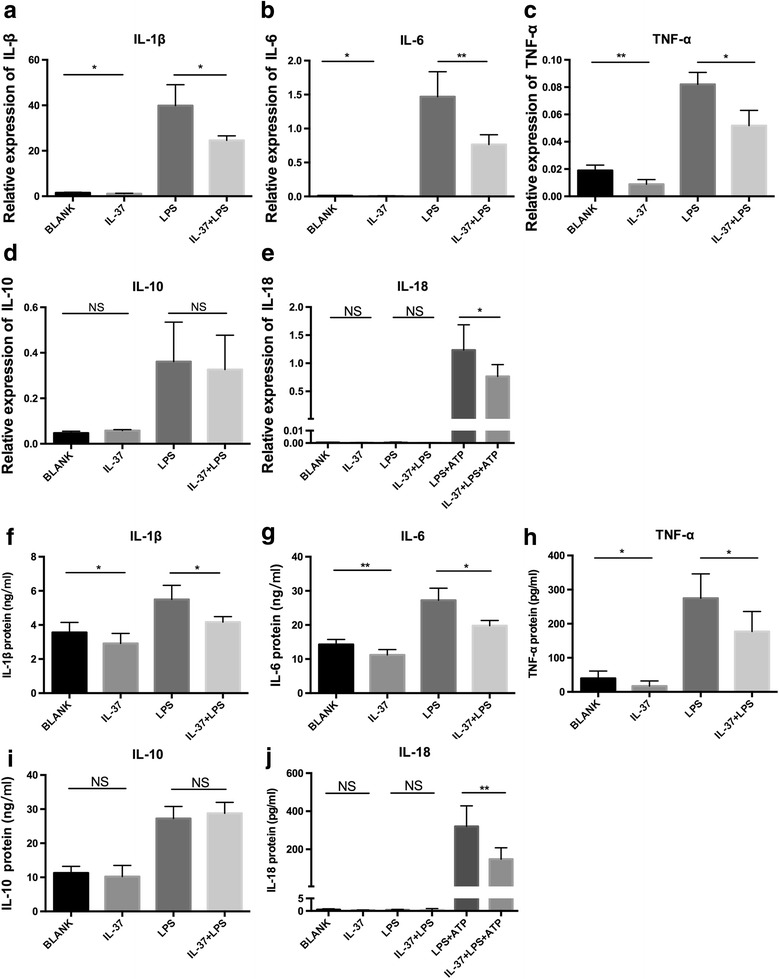
Interleukin (IL)-37 inhibits the expression of proinflammatory cytokines in peripheral blood mononuclear cells (PBMCs) of patients with adult-onset Still’s disease (AOSD). PBMCs of patients with AOSD (*n* = 10) were stimulated with human recombinant IL-37 (100 ng/ml) for 24 h, then incubated further with lipopolysaccharide (LPS) (1 μg/ml) for 4 h. **a**–**e** The total RNA was harvested for qRT-PCR analysis or incubated further with LPS (1 μg/ml) for 8 h. **f**–**j** The total protein was harvested for enzyme-linked immunosorbent assay analysis. The cells were further stimulated with 5 mM adenosine triphosphate (ATP) for 20 minutes for IL-18 messenger RNA (**e**) and protein (**j**) analysis. The data represent the mean ± SD. * *P* < 0.05, ** *P* < 0.01 by Student’s *t* test. *TNF-α* Tumor necrosis factor-α

IL-37 has been reported to be an inhibitor of innate immunity [31]. To assess whether IL-37 has a similar capacity to regulate the expression of inflammatory cytokines involved in the pathogenesis of AOSD, we evaluated the effects of IL-37 on LPS-induced cytokine expression in PBMCs of patients with AOSD. The PBMCs isolated from patients with AOSD were cultured in the presence or absence of rhIL-37 and further with LPS stimulation. The cell pellets and cultural supernatants were harvested for later qRT-PCR and ELISA analysis, respectively. We found that IL-37 significantly reduced spontaneous and LPS-induced IL-1β, IL-6, and TNF-α mRNA (Fig. 3a–c) and protein (Fig. 3f–h) expression, but the expression of IL-10 and IL-18 mRNA (Fig. 3d, e) and protein (Fig. 3i, j) was not clearly inhibited. However, we further incubated the PBMCs with 5 mM adenosine triphosphate (ATP) after IL-37 and LPS treatment, and we observed that IL-37 significantly reduced IL-18 mRNA (Fig. 3e) and protein (Fig. 3j) expression induced by ATP and LPS in patients with AOSD.

## Discussion

Previous studies have demonstrated that IL-37 acts as an immune mediator to restrain the inflammatory response of many inflammatory and autoimmune diseases. Yet, it is still not clear whether IL-37 is involved in the pathogenesis of AOSD. Our present results show that serum IL-37 levels were dramatically higher in patients with AOSD than in HC, and the levels of IL-37 were positively correlated with systemic score and laboratory features that represented AOSD disease activity. Serum IL-37 levels fell when disease activity was reduced upon follow-up of patients with AOSD. We confirmed the presence of high serum IL-37 levels in AOSD and demonstrated a possible role of IL-37 as a disease biomarker. In addition, we found that serum IL-37 protein levels were correlated with expression of proinflammatory cytokines IL-1β and IL-18 as well as anti-inflammatory cytokine IL-10. Moreover, serum IL-37 levels correlated significantly with clinical features of fever, skin rash, lymphadenopathy, splenomegaly, myalgia, and arthralgia in patients with AOSD. Consequently, it is likely that IL-37, as an important anti-inflammatory cytokine, may play a specific role in the systemic inflammation of AOSD. The disease course and prognosis for patients with AOSD may vary considerably [46]. Several studies demonstrate that IL-18, IFN-γ, IL-10, and IL-4 are associated with systemic AOSD, whereas IL-6, IL-17, and IL-23 are associated with arthritic AOSD [20, 47, 48]. Our studies showed that serum levels of IL-37 were comparable between patients who had a chronic articular pattern and those who had a systemic pattern (Additional file 1: Figure S2a) or between monophasic, polycyclic, and articular patterns (Additional file 1: Figure S2b). Because in our study patients with limited chronic articular AOSD were enrolled, further studies are needed to explore the role of IL-37 in different AOSD disease patterns.

Although the pathogenesis of AOSD is poorly understood, the published data have indicated that proinflammatory cytokines play an important role in the development of inflammation in AOSD. Previous studies demonstrated that serum levels of TNF-α, IL-6, IL-1β, and IL-18 are significantly elevated in patients with AOSD and that blocking these proinflammatory cytokines with antibodies or recombinant soluble receptor dramatically alleviated the disease severity in patients with AOSD [11, 12, 14, 18, 40, 43, 49, 50]. In this study, we observed higher serum levels of these cytokines in patients with AOSD (*see* Additional file 1: Figure S1). More importantly, we also found elevated serum IL-37 levels in patients with AOSD. Spearman’s correlation analysis showed that serum IL-37 levels were positively correlated with major proinflammatory cytokines such as IL-1β and IL-18 in patients with AOSD. IL-37 is expressed at low levels in PBMCs and DCs and is upregulated in an inducible manner. IL-37 is induced mainly in an inflammatory context. IL-1β, IL-18, TNF-α, IFN-γ, and transforming growth factor-β increase IL-37 synthesis, whereas IL-4 plus granulocyte-macrophage colony-stimulating factor inhibits IL-37 expression [31]. It has been demonstrated that the IL-37 mRNA and protein expression can be induced by TNF-α via activation of NF-κB and activator protein 1 signaling pathways in intestinal epithelial cells [51]. These results indicate that these proinflammatory cytokines act as positive feedback loops for upregulation of IL-37 production. Therefore, it is reasonable to explain the correlation between higher disease activity and higher IL-37 expression levels in AOSD, and an increase in IL-37 levels may result from an excessive inflammatory response in AOSD.

Moreover, our results show that serum IL-37 levels were positively correlated with the anti-inflammatory cytokine IL-10. Previous studies showed that upregulation of genes encoding negative regulators of innate immunity, such as IL-10 [39], IL-1 receptor antagonist, and SOCS3 [52], were elevated in patients with active AOSD, and IL-10 showed a trend similar to that of the AOSD disease activity [53]. These anti-inflammatory responses were presumably an attempt to quell aberrant inflammation in active AOSD [54]. The activation of anti-inflammatory mechanisms was also supported by the M2 polarization of macrophages of patients with systemic juvenile idiopathic arthritis, who shared clinical similarities with patients with AOSD. Contrary to classically activated macrophages (M1) that were highly proinflammatory, M2 macrophages were intended to resolve inflammation, perform scavenger functions, and promote tissue repair. The M2 polarization could result from stimulation by anti-inflammatory cytokines such as IL-4, IL-37, or IL-10 [55]. In our study, serum IL-37 levels in patients with AOSD were positively correlated with IL-10; thus, we speculate that M2 macrophages may have a role in the pathogenesis of AOSD. Several other studies showed that the expression level of IL-10 in PBMCs was increased after rhIL-37 treatment, which is different from our results [56–58]. In IL37-tg mice subjected to a model of inflammatory colitis, there was also an increase in IL-10 production by colon tissue; however, the protective effects of IL-37 were shown to be IL-10-independent because antibody blockade of the IL-10 receptor did not abrogate the effects. In mice subjected to inflammatory arthritis, the efficacy of treatment with recombinant IL-37 was also unrelated to induction of IL-10 [27]. We speculate that inflammation signaling not only exacerbated the inflammatory response in the pathogenesis of AOSD but also promoted the expression of anti-inflammatory cytokines such as IL-37 and IL-10 to limit excessive inflammation in AOSD through specific and distinct anti-inflammatory properties [29].

In recent reports, IL-37 was shown to be a negative mediator reducing proinflammatory cytokine production in inflammatory diseases. Nold et al. found that inhibition of endogenous IL-37 with small interfering RNAs in human PBMCs increased the production of IL-1β, IL-6, and TNF-α [31], suggesting that IL-37 is crucial for inflammation control. In addition, IL-37 significantly suppresses the production of proinflammatory cytokines and the activation of DCs [31, 32, 59]. To further explore the effects of IL-37 on the proinflammatory cytokines that are responsible for the pathogenesis of AOSD, the rhIL-37 protein was used to stimulate PBMCs from patients with AOSD. In our study, we showed that rhIL-37 remarkably attenuated spontaneous and LPS-induced TNF-α, IL-6, and IL-1β expression in PBMCs from patients with AOSD. Although the inhibition of proinflammatory cytokine signaling pathways by IL-37 remains elusive, Nold et al. revealed that IL-37 can reduce the expression of STAT3 [31], whereas STAT3 has been reported to be closely related to AOSD pathogenesis [60]. We suspect that IL-37 may attenuate the production of proinflammatory cytokines through regulating several critical signal transducers, such as STAT3, to smother the excessive inflammation in patients with AOSD.

## Conclusions

In the present study, we showed that the upregulation of IL-37 was positively correlated with AOSD disease activity, indicating its involvement in AOSD pathogenesis, and it may become a novel disease activity biomarker. Our results derived from the cell-based functional assay suggest that IL-37 may participate in the course of AOSD through the reduction of proinflammatory cytokines. Further studies are required to confirm the regulatory mechanism of IL-37 in the pathogenesis of AOSD and extend the present findings.

## Additional file

Additional file 1: **Figure S1.** Comparison of serum cytokines between AOSD and HC. Serum IL-1β (**a**), IL-1Rα (**b**), TNF-α (**c**), soluble tumor necrosis factor receptor (**d**), IL-6 (**e**), IL-18 (**f**), IL-17 (**g**), and IL-10 (**h**) protein levels among patients with AOSD with active and inactive disease activity (active [*n* = 41] versus inactive [*n* = 21]) as well as HC (*n* = 50) were determined by ELISA. Each symbol represents an individual patient with AOSD and an HC. *Horizontal lines* indicate median values. The data represent the mean ± SD. *** *P* < 0.001 by Student’s *t* test. **Figure S2.** Comparison of serum IL-37 levels between patients with AOSD with different disease patterns. (**a**) The serum IL-37 levels in 59 patients with AOSD with a systemic course and 3 with an articular course. (**b**) The serum IL-37 levels in 39 patients with AOSD with a monophasic course, 20 with a polycyclic course and 3 with an articular course. Data are expressed as the mean ± SD. The Mann-Whitney *U* test was used to perform the statistical analysis. (ZIP 2677 kb)

## Abbreviations

ALT: Alanine transaminase
ANA: Antinuclear antibody
AOSD: Adult-onset Still’s disease
AS: Ankylosing spondylitis
AST: Aspartate transaminase
ATP: Adenosine triphosphate
CRP: C-reactive protein
DC: Dendritic cell
ELISA: Enzyme-linked immunosorbent assay
ESR: Erythrocyte sedimentation rate
GAPDH: Glyceraldehyde 3-phosphate dehydrogenase
HC: Healthy control subject
rhIL-37: Recombinant human interleukin-37
IFN-γ: Interferon-γ
IL: Interleukin
IL37-tg: Interleukin-37-transgenic
LPS: Lipopolysaccharide
mRNA: Messenger RNA
NF-κB: Nuclear factor-κB
PBMC: Peripheral blood mononuclear cell
RA: Rheumatoid arthritis
RF: Rheumatoid factor
sDMARD: Synthetic disease-modifying antirheumatic drug
SLE: Systemic lupus erythematosus
TLR: Toll-like receptor
TNF-α: Tumor necrosis factor-α
