# Supplementary figures and images for: Interleukin-37 is increased in adult-onset Still’s disease and associated with disease activity
Source: Arthritis Res Ther. 2018 Mar 22;20:54. doi: 10.1186/s13075-018-1555-6 (PMC5863797; doi:10.1186/s13075-018-1555-6)

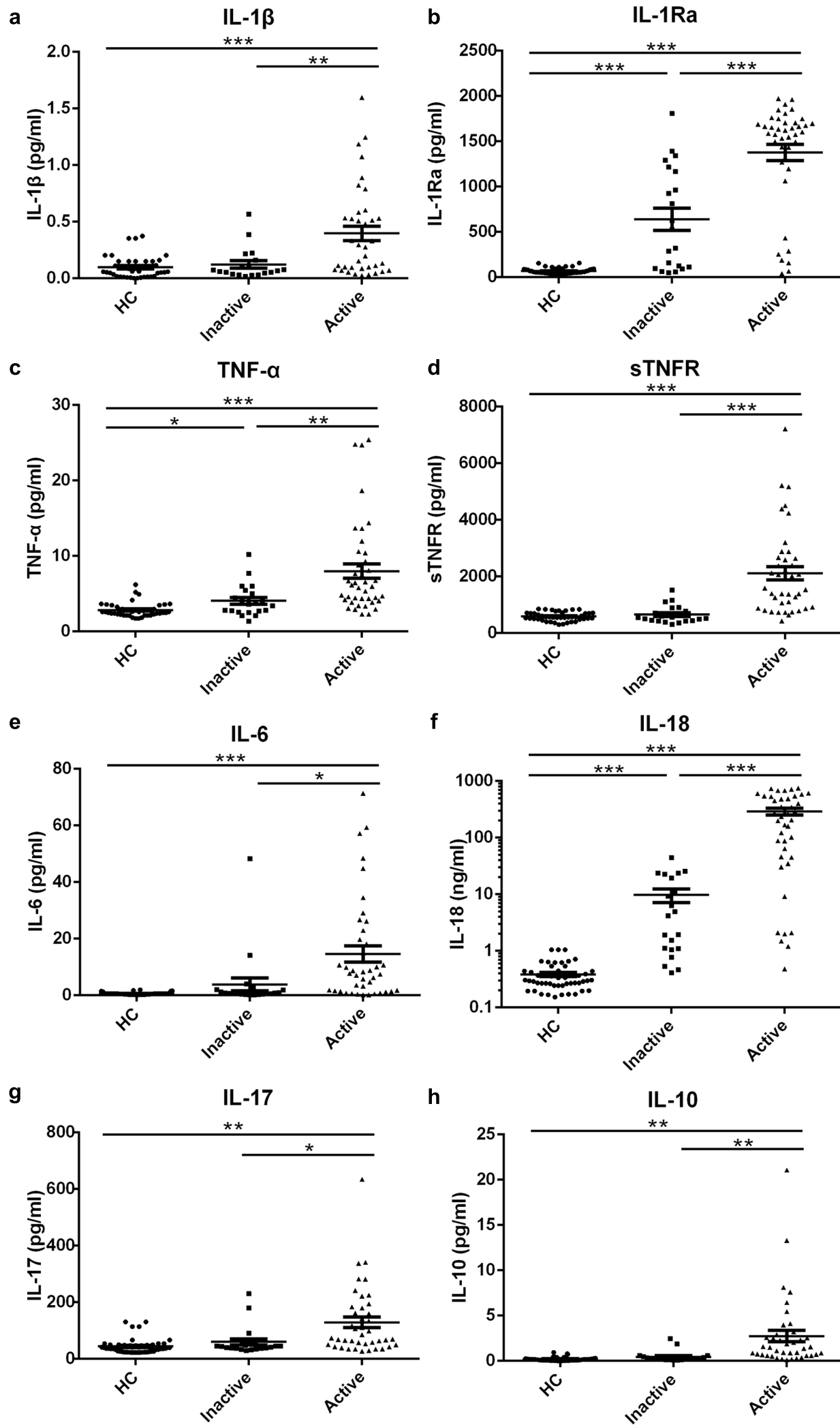

Supplement: Supplementary file 1 — Figure S1. Comparison of serum cytokines between AOSD and HC. Serum IL-1β (a), IL-1Rα (b), TNF-α (c), soluble tumor necrosis factor receptor (d), IL-6 (e), IL-18 (f), IL-17 (g), and IL-10 (h) protein levels among patients with AOSD with active and inactive disease activity (active [n = 41] versus inactive [n = 21]) as well as HC (n = 50) were determined by ELISA. Each symbol represents an individual patient with AOSD and an HC. Horizontal lines indicate median values. The data represent the mean ± SD. *** P < 0.001 by Student’s t test. Figure S2. Comparison of serum IL-37 levels between patients with AOSD with different disease patterns. (a) The serum IL-37 levels in 59 patients with AOSD with a systemic course and 3 with an articular course. (b) The serum IL-37 levels in 39 patients with AOSD with a monophasic course, 20 with a polycyclic course and 3 with an articular course. Data are expressed as the mean ± SD. The Mann-Whitney U test was used to perform the statistical analysis. (ZIP 2677 kb) [file 13075_2018_1555_MOESM1_ESM.zip › Fig S1.pdf]

a

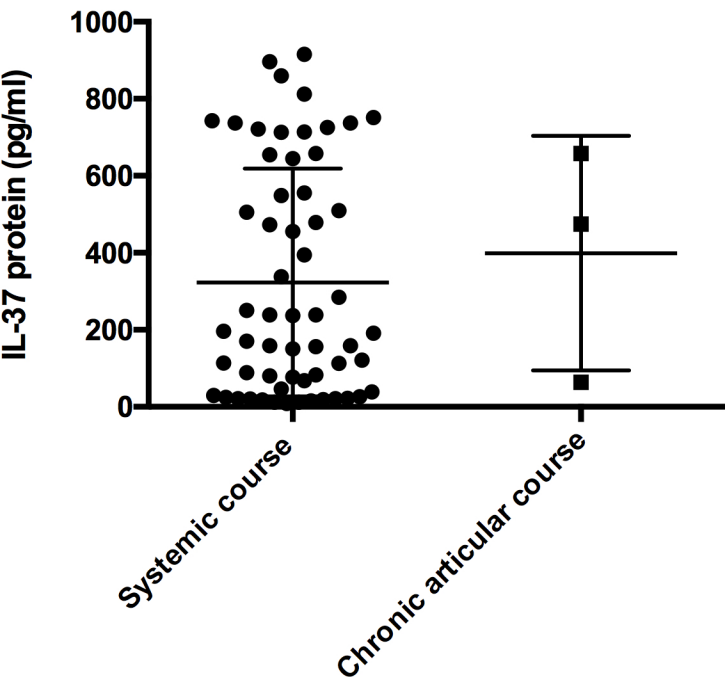

b

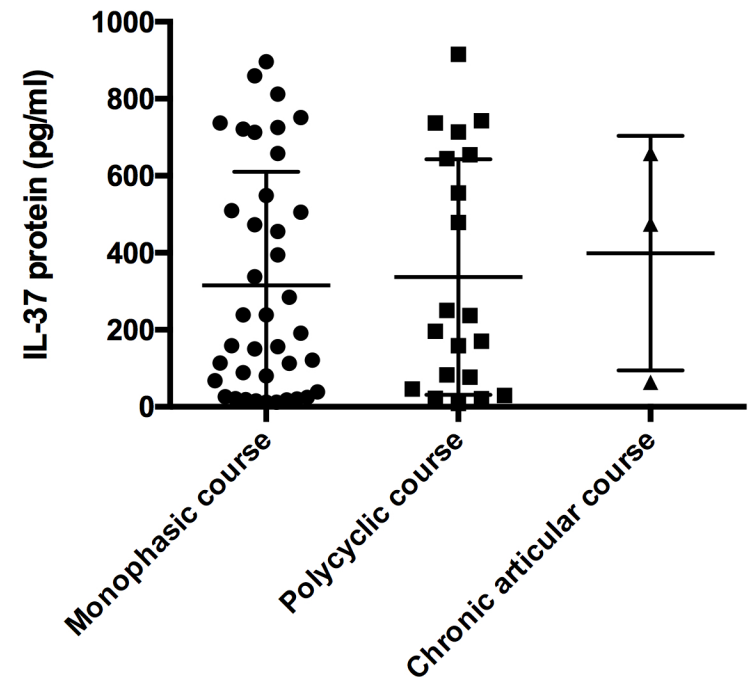

Supplement: Supplementary file 1 — Figure S1. Comparison of serum cytokines between AOSD and HC. Serum IL-1β (a), IL-1Rα (b), TNF-α (c), soluble tumor necrosis factor receptor (d), IL-6 (e), IL-18 (f), IL-17 (g), and IL-10 (h) protein levels among patients with AOSD with active and inactive disease activity (active [n = 41] versus inactive [n = 21]) as well as HC (n = 50) were determined by ELISA. Each symbol represents an individual patient with AOSD and an HC. Horizontal lines indicate median values. The data represent the mean ± SD. *** P < 0.001 by Student’s t test. Figure S2. Comparison of serum IL-37 levels between patients with AOSD with different disease patterns. (a) The serum IL-37 levels in 59 patients with AOSD with a systemic course and 3 with an articular course. (b) The serum IL-37 levels in 39 patients with AOSD with a monophasic course, 20 with a polycyclic course and 3 with an articular course. Data are expressed as the mean ± SD. The Mann-Whitney U test was used to perform the statistical analysis. (ZIP 2677 kb) [file 13075_2018_1555_MOESM1_ESM.zip › Fig S2.pdf]
